# Molecular characteristics of global β-lactamase-producing *Enterobacter cloacae* by genomic analysis

**DOI:** 10.1186/s12866-022-02667-y

**Published:** 2022-10-21

**Authors:** Jincao Hu, Jia Li, Chang Liu, Yan Zhang, Hui Xie, Chuchu Li, Han Shen, Xiaoli Cao

**Affiliations:** 1 Department of Laboratory Medicine, Nanjing Drum Tower Hospital, the Affiliated Hospital of Nanjing University Medical School, Zhongshan Road 321, GulouJiangsu Province, Nanjing, People’s Republic of China; 2 Department of Acute Infectious Disease Control and Prevention, Jiangsu Provincial Center for Disease Control and Prevention, Nanjing, China

**Keywords:** *Enterobacter cloacae*, β-lactamase, Sequence type, Carbapenem-hydrolyzing β-lactamase

## Abstract

**Objective:**

To analyze the characteristics of global β-lactamase-producing *Enterobacter cloacae* including the distribution of β-lactamase, sequence types (STs) as well as plasmid replicons.

**Methods:**

All the genomes of the *E. cloacae* were downloaded from GenBank. The distribution of β-lactamase encoding genes were investigated by genome annotation after the genome quality was checked. The STs of these strains were analyzed by multi-locus sequence typing (MLST). The distribution of plasmid replicons was further explored by submitting these genomes to the genome epidemiology center. The isolation information of these strains was extracted by Per program from GenBank.

**Results:**

A total of 272 out of 276 strains were found to carry β-lactamase encoding genes. Among them, 23 varieties of β-lactamase were identified, *bla*_CMH_ (*n* = 130, 47.8%) and *bla*_ACT_ (*n* = 126, 46.3%) were the most predominant ones, 9 genotypes of carbapenem-hydrolyzing β-lactamase (CHβLs) were identified with *bla*_VIM_ (*n* = 29, 10.7%) and *bla*_KPC_ (*n* = 24, 8.9%) being the most dominant ones. In addition, 115 distinct STs for the 272 ß-lactamase-carrying *E. cloacae* and 48 different STs for 106 CHβLs-producing *E. cloacae* were detected. ST873 (*n* = 27, 9.9%) was the most common ST. Furthermore, 25 different plasmid replicons were identified, IncHI2 (*n* = 65, 23.9%), IncHI2A (*n* = 64, 23.5%) and IncFII (*n* = 62, 22.8%) were the most common ones. Notably, the distribution of plasmid replicons IncHI2 and IncHI2A among CHβLs-producing strains were significantly higher than theat among non-CHβLs-producing strains (*p* < 0.05).

**Conclusion:**

Almost all the *E. cloacae* contained β-lactamase encoding gene. Among the global *E. cloacae*, *bla*_CMH_ and *bla*_ACT_ were main *bla*_AmpC_ genes. *Bla*_TEM_ and *bla*_CTX-M_ were the predominant ESBLs. *Bla*_KPC_, *bla*_VIM_ and *bla*_NDM_ were the major CHβLs. Additionally, diversely distinct STs and different replicons were identified.

**Supplementary Information:**

The online version contains supplementary material available at 10.1186/s12866-022-02667-y.

## Introduction

*Enterobacter (E. cloacae)* belongs to facultative anaerobic Gram-negative bacilli, grouping into the *E. cloacae* complex group, the family *Enterobacterale* [1]***.*** Generally, such bacteria colonize soil and water as well as the animal and human gut, representing one of the most leading species described in clinical infections, particularly in vulnerable patients [2]. It has been reported that *E. cloacae* is frequently associated with a multidrug resistance (MDR) phenotype, due to the inducible overproducing AmpC β-lactamases and acquisition of numerous genetic mobile elements containing resistance [3]. More worrisome, the production of carbapenem-hydrolyzing β-lactamase (CHβLs) rendering ineffective almost all β-lactams families have been continually acquired, resulting in the production of super-resistant bacteria carbapenem-resistant *Enterobacter cloacae* (CREL) [4].

β-lactamase is a predominant resistance determinant for β-lactam antibiotics in *E. cloacae*. To date, there are two classification schemes for β-lactamases, the more groupings in clinical laboratory generally correlate with broadly based molecular classification, where β-lactamases are divided into class A, B, C and D enzymes based on the amino acid sequence [5]. Currently, the most problematic enzymes are plasmid-mediated AmpC β-lactamases (pAmpCs) with *bla*_ACT-like_ *ampC* genes being highly prevalent [6], extended-spectrum β-lactamases (ESBLs) with *bla*_SHV_ and *bla*_CTX-M_ being widely distributed [7], and CHβLs, all of which are challenging antibiotic effectiveness.

Globally, *bla*_CHβLs_ such as *bla*_KPC_ (class A), *bla*_NDM/VIM/IMP_ (class B) and *bla*_OXA-48_ (class D) are of grave clinical concern and proliferating [8]. It was reported that *bla*_NDM-1_ and *bla*_NDM-5_ were the main *bla*_CHβLs_, ST93, ST171 and ST145 was the predominant sequence types (STs) for CREL in a tertiary Hospital in Northeast China during 2010–2019 [9]. Whereas in Japan, *bla*_IMP-1_ was the dominant *bla*_CHβLs_ conferring carbapenem resistance [10], and *bla*_VIM_ was the main *bla*_CHβLs_ in France between 2015–2018 [11]. However, the whole distribution of β-lactamase among global *E. cloacae* is unclear, and information on the clones of *E. cloacae* spreading internationally remains unknown. As we know that plasmids play an important role in horizontal gene transfer of antimicrobial resistance genes (ARG), and the identification of replicon types is helpful to analyze plasmid characteristics. Further, the association between plasmid replicons and different resistant determinants is essential to understand the role of plasmids in transmission of ARG [12]. For instance, IncN plasmids have been reported to be the predominant replicon types for *bla*_IMP-4_-carrying strains [13], however, the prevalence of plasmid replicons among these bacteria were unknown. Notably, the association between IncIγ plasmid encoding *bla*_CMY-2_ ß-lactamase and the international ST19 was observed in multidrug-resistant *Salmonella Typhimurium* [14]. Whether or not this phenomenon could be observed in *E. cloacae* needs to be confirmed.

With the extensive use and development of antibacterial drugs, β-lactamases have evolved rapidly. Meanwhile, due to the rapid development of whole-genome sequencing (WGS) technology, the number of sequenced bacterial genomes has grown enormously, new β-lactamase variants continue to be described. As a common opportunistic pathogen [15], the information on the distribution of β-lactamase among *E. cloacae* was limited.

In this study, we first explored the distribution of β-lactamase including pAmpCs, *bla*_ESBLs_ and *bla*_CHβLs_ among *E. cloacae* isolates based on a global database. For β-lactamase positive strains, the sequence types (STs) and the distribution of plasmid replicons were further investigated. Furthermore, the prevalent characteristics of β-lactamase-producing *E. cloacae* were analyzed.

## Materials and methods

### Acquisition of *E. cloacae* genomes and strain information

A total of 296 *E. cloacae* genomes were downloaded in batches from NCBI using Aspera software on 16^th^, Dec 2021 [16]. The genomic quality of these 296 strains was further filtered by Checkm and Quast software [17, 18]. The high-quality genome was defined as “completeness > 90% and containment < 5%”. Meanwhile, the quantity of contigs is required to be “ ≤ 500, and N50 ≥ 40,000”. Twenty genomes that did not meet the above conditions were filtered out. The investigated strains were collected from different years shown in Figure S1A, the collected dates of 58 strains were “blank” meaning that the information was missing. These strains were submitted by 32 countries, mainly from USA (*n* = 58), France (*n* = 30), United Kingdom (*n* = 27), China (*n* = 24), Japan (*n* = 18), Singapore (*n* = 13) and Nigeria (*n* = 12), other countries were also involved (Figure S1B). The countries of 26 strains remained unknown. Notably, 158 out of 272 strains were hosted by Homo sapiens (*n* = 158, 58.1%), mainly from gastrointestinal tract (*n* = 57, 21.0%).

### Investigation of β-lactamase among global *E. cloacae*

To avoid differences in genome gene prediction by different annotation methods. All the 276 genomes were annotated by Prokka software [19], which is a fast prokaryotic genome annotation software. All the strains containing β-lactamase encoding genes were further analyzed.

### Analysis on the sequence type of β-lactamase carrying *E. cloacae*

The self-made Perl program was used to extract the nucleotide coding sequence of genes from each genome sequence file (GBK format) [20]. The allele sequences and allelic profiles of 7 conserved genes of *E. cloacae* were downloaded from website https://pubmlst.org/. The sequence of the genome was set as “query”, the seven conserved gene sequence files were set as “subject “(database). Blastn alignment analysis was then implemented between query and subject. The thresholds set were as follows: E-value = 1e-5, identity = 100%, matching length = subject gene length.

### Investigation of plasmid replicons among ß-lactamase positive *E. cloacae*

To analyze the distribution of plasmid replicons among β-lactamase-carrying *E. cloacae.* The genomes were submitted into the website and PlasmidFinder (https://cge.cbs.dtu.dk/services/PlasmidFinder/) was used to analyze the presence of plasmid replicons (Identity: 90%; Coverage: 90%).

### Statistical analysis

The differences on the distribution of major resistant determinants and plasmid replicons among *bla*_CHβLs_-carrying strains and strains without *bla*_CHβLs_ was analyzed by Chi-square test. Distribution difference on resistant determinants and plasmid replicons among all the β-lactamase-producing and among the *bla*_CHβLs_-carrying strains were checked by McNemar test. The distribution rates were statistically different when *p* value was less than 0.05.

## Results

### The distribution of β-lactamase among global *E. cloacae*

In total, 272 out of 276 strains were found to carry β-lactamase encoding genes. There were 23 varieties of ß-lactamase being found, *bla*_CMH_ (*n* = 130, 47.8%) and *bla*_ACT_ (*n* = 126, 46.3%) were the most predominant ones. Other ß-lactamase encoding genes included *bla*_TEM_ (*n* = 90, 33.1%), *bla*_OXA_ (*n* = 51,18.8%), *bla*_CTX-M_ (*n* = 48, 17.6%), *bla*_VIM_ (*n* = 29, 10.7%), *bla*_KPC_ (*n* = 24, 8.8%), *bla*_SHV_ (*n* = 23, 8.5%), *bla*_NDM_ (*n* = 22, 8.1%), *bla*_IMI_ (*n* = 17, 6.3%), *bla*_MIR_ (*n* = 11, 4.0%), *bla*_LAP-2_ (*n* = 10, 3.7%), *bla*_IMP_ (*n* = 7, 2.6%), *bla*_DHA_ (*n* = 7, 2.6%), *bla*_GES_ (*n* = 4, 1.5%), *bla*_CMY_ (*n* = 3,1.1%), *bla*_FOX-5_ (*n* = 3,1.1%), *bla*_VEB-3_ (*n* = 2, 0.7%), *bla*_NMC-A_ (*n* = 2, 0.7%), *bla*_CARB_ (*n* = 2, 0.7%), *bla*_FLC-1_ (*n* = 1, 0.4%), *bla*_ORN-1_ (*n* = 1, 0.4%) and *bla*_SCO-1_ (*n* = 1, 0.4%).

**Fig. 1:**
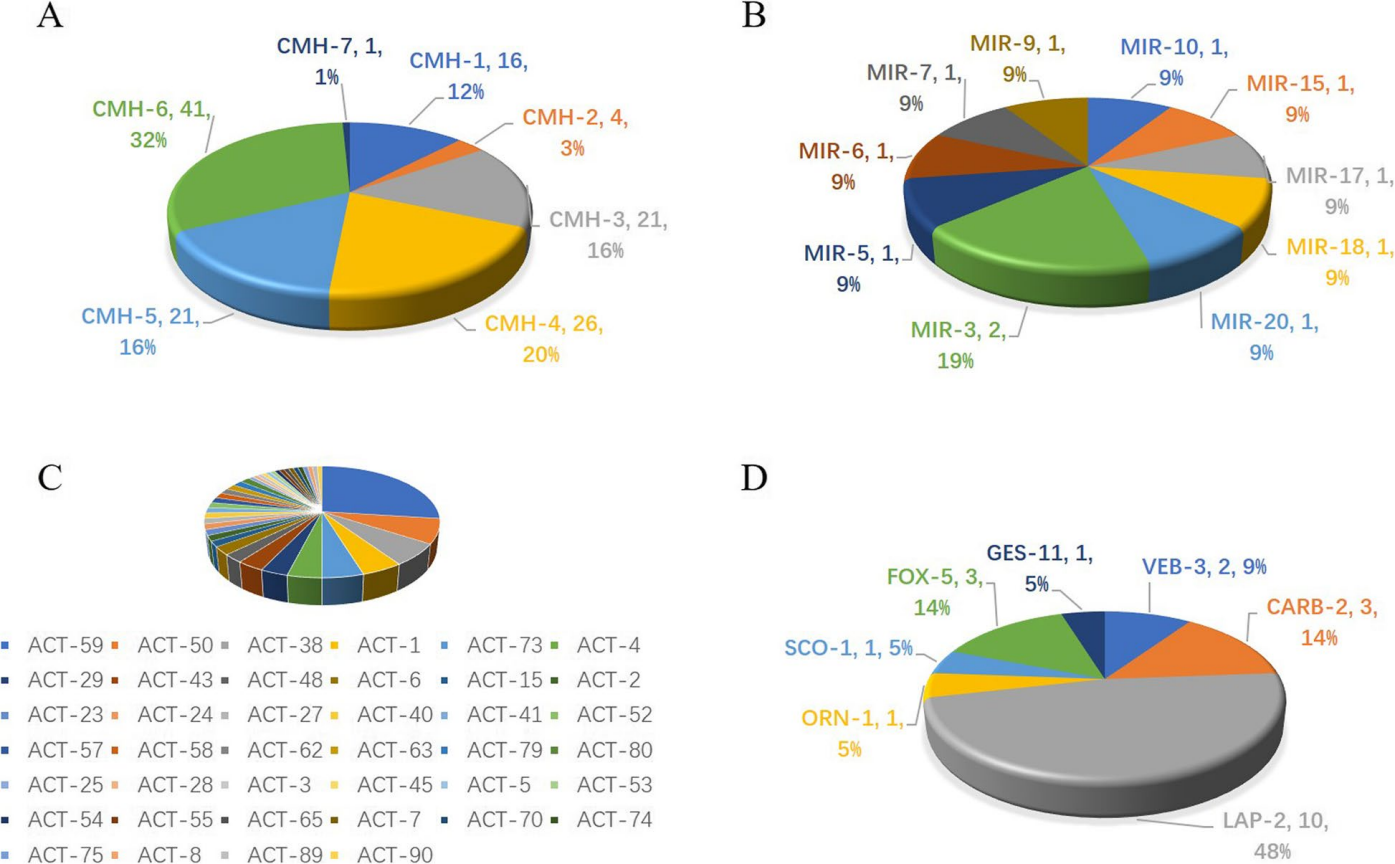
Variants of the predominant plasmid-mediated AmpC β-lactamases (pAmpCs) among *Enterobacter cloacae*. 1A, variants of *bla*_CMH_; 1B, variants of *bla*_MIR_; 1C, variants of *bla*_ACT_; 1D, variants of other pAmpCs

**Fig. 2:**
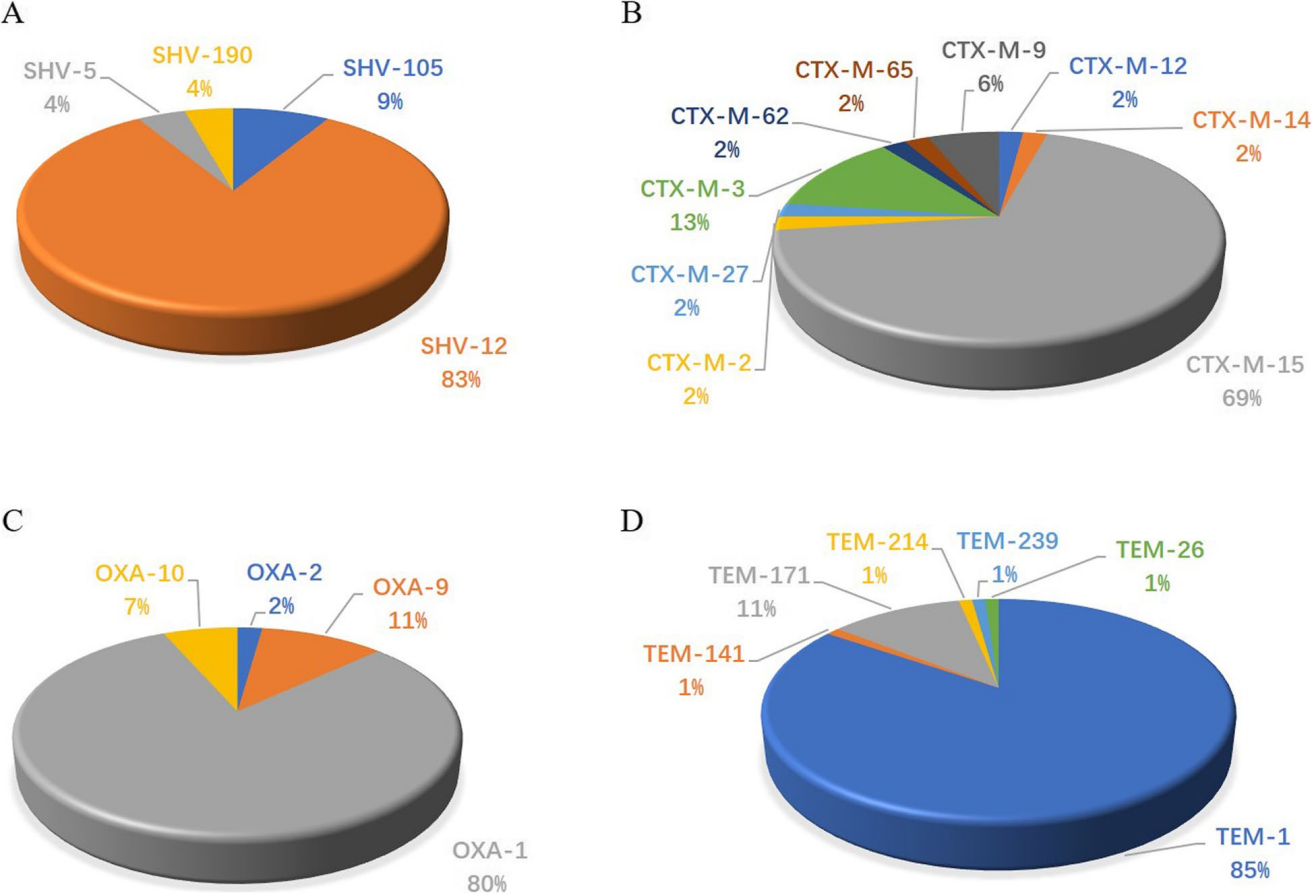
Variants of the predominant extended-spectrum β-lactamases (ESBLs) among *Enterobacter cloacae*. 2A, Variants of *bla*_SHV_; 2B, Variants of *bla*_CTX-M_; 3B, Variants of *bla*_OXA_; *4B,* Variants of *bla*_TEM_

In detail, the variants of pAmpCs including *bla*_CMH_, *bla*_ACT_ and *bla*_MIR_ were shown in Fig. 1, with *bla*_CMH-6_ (*n* = 41, 15.1%) and *bla*_ACT-59_(*n* = 34, 12.5%) being the most frequent ones. Multiple variants of *bla*_ESBLs_ including *bla*_CTX_, *bla*_TEM_, *bla*_OXA_ and *bla*_SHV_ were also found (Fig. 2). Among them, *bla*_CTX-M-15_ (*n* = 33, 12.1%) and *bla*_SHV-12_ (*n* = 19, 7.0%) were the most common ones.

**Fig. 3:**
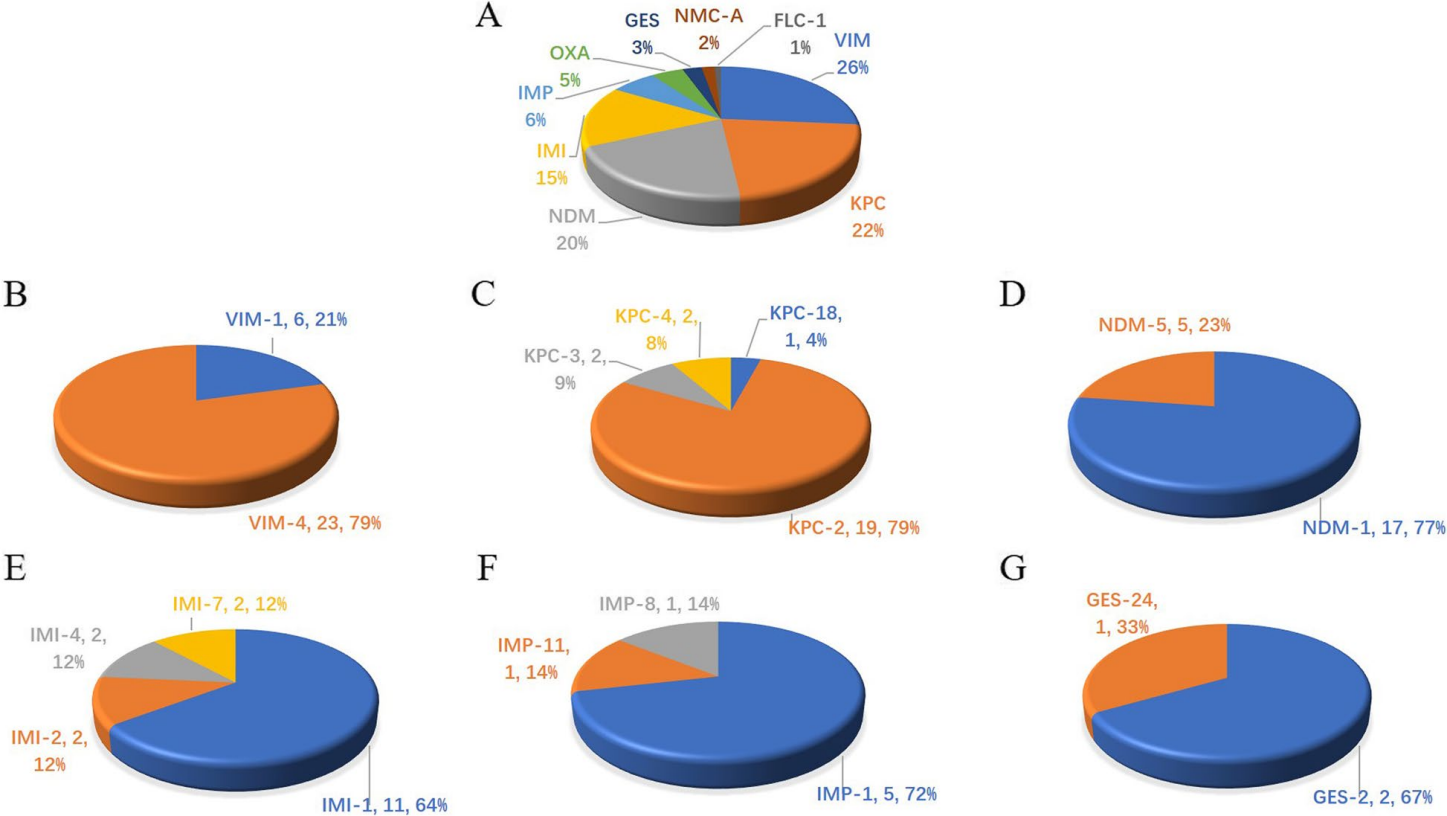
Variants of the predominant carbapenem-hydrolyzing β-lactamase (CHβLs) among *Enterobacter cloacae.* 3A, carbapenemase detected in this study. 3B, Variants of *bla*_VIM_; 3C, Variants of *bla*_KPC_; 3D, Variants of *bla*_NDM_; 3E, Variants of *bla*_IMI_; 3F, Variants of *bla*_IMP_; 3G, Variants of *bla*_GES_

Overall, 9 genotypes of *bla*_CHβLs_ including *bla*_NDM_, *bla*_IMP_, *bla*_OXA_, *bla*_KPC_, *bla*_VIM_, *bla*_FLC-1_, *bla*_NMC-A_, *bla*_GES_ and *bla*_IMI_ were found among 106 strains (Fig. 3). Besides the *bla*_CHβLs_ in the Fig. 3, other ones including *bla*_OXA-48_ (*n* = 3, 2.9%) and *bla*_OXA-181_ (*n* = 2, 1.9%), *bla*_NMC-A_ (*n* = 2, 1.9%) and *bla*_FLC-1_ (*n* = 1, 1.0%) were also identified.

The distribution of *bla*_ACT_, *bla*_SHV_ and *bla*_TEM_ were obviously higher among *bla*_CHβLs_-carrying *E. cloacae* comparing to the prevalence of these genes among the strains without *bla*_CHβLs_ (*p* < 0.05), whereas *bla*_CMH_ and oxacillin-hydrolyzing-*bla*_OXA_ were much more prevalent among *E. cloacae* strains without *bla*_CHβLs_ than *bla*_CHβLs_-carrying ones (*p* < 0.05) (Table 1).

**Table 1:** The differences on the distribution of resistant determinants among *bla*_CHβLs_ positive and *bla*_CHβLs_ negtive *Enterobacter cloacae*

	*bla*_CHβLs_ positive strains (*n* = 106)	*bla*_CHβLs_ negative strains (*n* = 166)	Chi-square value	*P* value
*bla*_CMH_ (*n* = 130)	41 (38.7%)	89 (53.6%)	5.873	0.016
*bla*_ACT_ (*n* = 126)	60 (56.7%)	66 (39.8%)	7.382	0.007
*bla*_OXA_ (*n* = 51)	3 (2.8%)	27 (16.3%)	10.569^a^	0.001
*bla*_CTX-M_ (*n* = 48)	18 (17.0%)	29 (17.5%)	0.011	0.917
*bla*_SHV_ (*n* = 23)	16 (15.1%)	5 (3.0%)	13.255	0.000
*bla*_TEM_ (*n* = 90)	61 (57.5%)	29 (17.5%)	46.932	0.000

*CHβLs* Carbapenem-hydrolyzing β-lactamase

^a^ Continuity correction

### The sequence types of β-lactamase-carrying *E. cloacae*

**Fig. 4:**
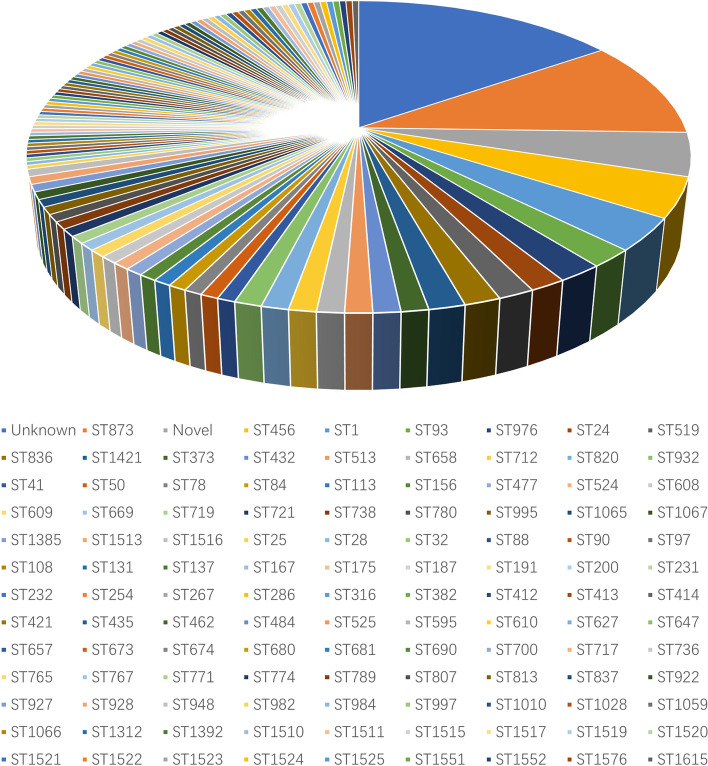
The sequence types of 272 β-lactamase-producing *Enterobacter cloacae*

Totally, there were 115 distinct STs for the 272 β-lactamase-carrying *E. cloacae* (Fig. 4). ST873 (*n* = 27, 23.5%) was the most frequent one followed by ST456 (*n* = 11, 9.6%). ST1 (*n* = 9, 7.8%), ST93 (*n* = 5, 4.3%) and ST976 (*n* = 5, 4.3%) were less common. The STs of 41 strains remained unknown and 12 strains belonged to novel STs. Other 110 STs were scattered (Fig. 4).

**Fig. 5:**
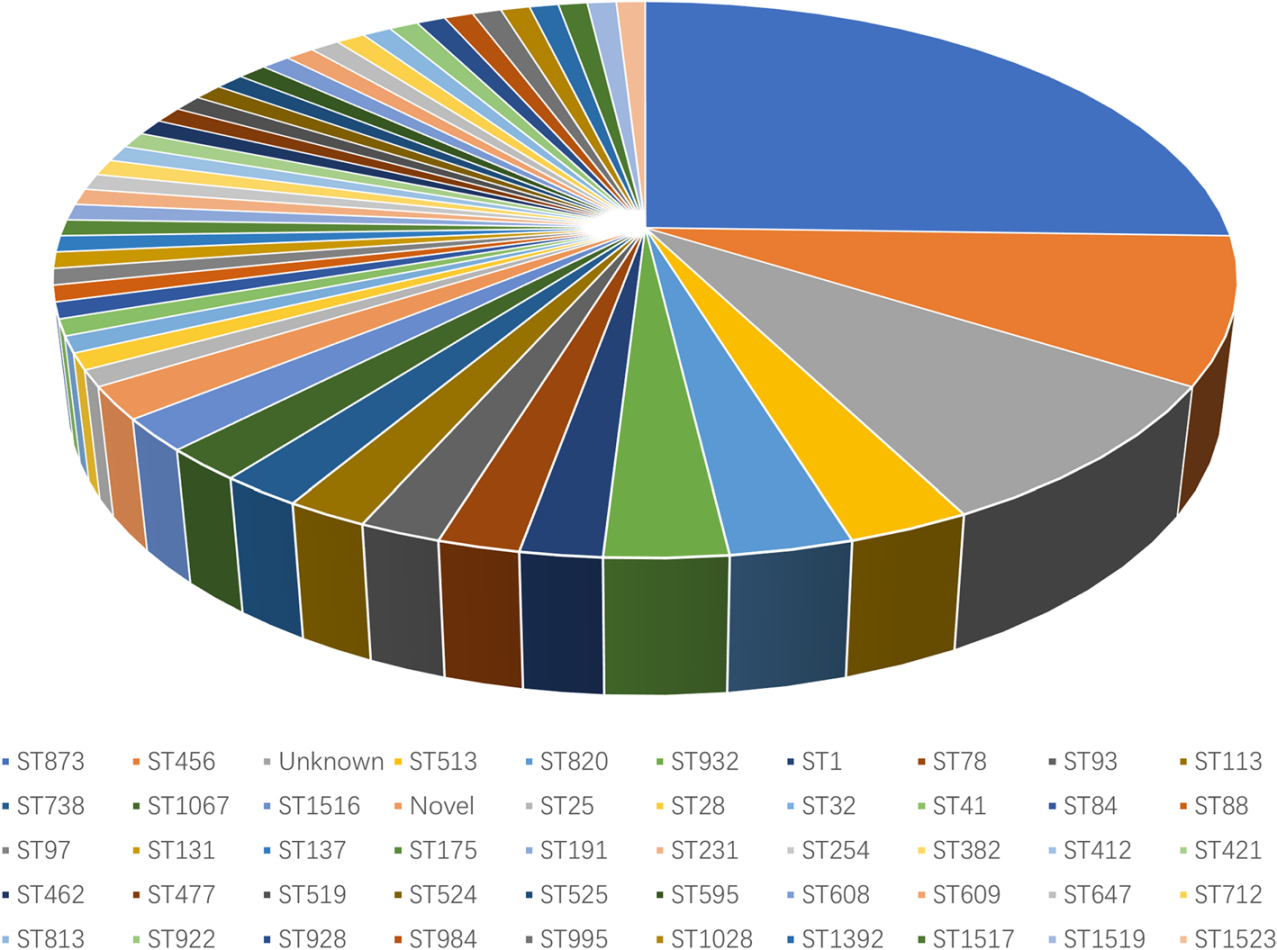
The sequence types of 106 carbapenem-hydrolyzing β-lactamase-producing *Enterobacter cloacae*

**Fig. 6:**
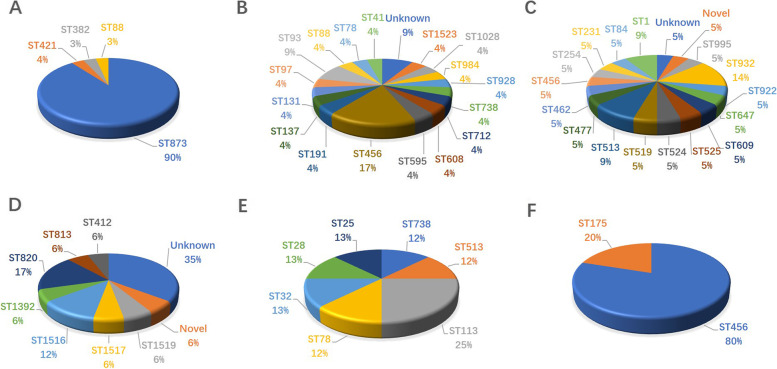
The sequence types of dominant carbapenem-hydrolyzing β-lactamase-producing *Enterobacter cloacae.* 6A, the sequence types (STs) of *bla*_VIM_-carrying strains; 6B, The STs of *bla*_KPC_-carrying strains; 6C, STs of *bla*_NDM_-carrying strains; 6D, STs of *bla*_IMI_-carrying strains; 6E, STs of *bla*_IMP_-carrying strains; 6F, STs of *bla*_OXA_-carrying strains

Furthermore, 48 different STs were identified for *bla*_CHβLs_-carrying *E. cloacae* (Fig. 5). And ST873 (*n* = 27, 25.7%) and ST456 (*n* = 11,10.5%) was the most common ones. Diverse STs were identified for *bla*_CHβLs_-carrying *E. cloacae* (Fig. 6). Interestingly, all the 23 *bla*_VIM-4_-carrying *E. cloacae*, and 3 out of 6 *bla*_VIM-1_- carrying *E. cloacae* isolates were assigned into ST873 (Fig. 6A). Whereas 19 *bla*_KPC-2_ ones were assigned to 13 STs (Fig. 6B), and 17 *bla*_NDM-1_ ones were assigned into 14 STs (Fig. 6C). Furthermore, 9 distinct STs for 17 *bla*_IMI_- carrying strains (Fig. 6D), 7 different STs for 7 *bla*_IMP_-carrying ones (Fig. 6E) and 2 STs for 5 strains carrying carbapenem-hydrolyzing *bla*_OXA_ (Fig. 6F) were identified. Of note, 27 out of 34 *bla*_ACT-59_ were found to be carried by ST873 strains.

### The plasmid replicons of CHβLs-carrying *E. cloacae*

Totally, 25 different plasmid replicons were identified. IncHI2 (*n* = 65, 23.9%), IncHI2A (*n* = 64, 23.5%) and IncFII (*n* = 62, 22.8%) were the most common ones followed by IncCol (*n* = 48, 17.6%), IncFII (*n* = 41, 15.1%) and IncR (*n* = 28, 10.3%). IncFIA (*n* = 20, 7.4%), IncN (*n* = 18, 6.6%), IncX3 (*n* = 12, 4.4%), IncC (*n* = 8, 2.9%), IncHI1B (*n* = 8, 2.9%), IncM1 (*n* = 7, 2.6%), IIncHI1A (*n* = 6, 2.2%), IncP6 (*n* = 5, 1.8%), pKPC-CAV1193 (*n* = 4, 1.5%), IncQ1 (*n* = 3, 1.1%), IncL (*n* = 3, 1.1%), IncX5 (*n* = 2, 0.7%), IncX4 (*n* = 1, 0.4%), IncM2 (*n* = 1, 0.4%), IncN2 (*n* = 1, 0.4%), IncP1 (*n* = 1, 0.4%), IncA (*n* = 1, 0.4%), repA (*n* = 1, 0.4%) and repB (*n* = 1, 0.4%) were also found. It was worth mentioning that no plasmid replicons were found among 97 strains, 62 (22.8%) out of which only contained one *bla*_CMH_, 21 (7.7%) ones carried *bla*_CHβLs_.

Notably, the prevalence of replicons IncHI2 and IncHI2A among *bla*_CHβLs_ -carrying strains were significantly higher than that among the strains without *bla*_CHβLs_ (*p* < 0.05), whereas no significant difference on the prevalence of plasmid replicons IncCOI, IncFII, IncFIB and IncR among these two groups were observed (Table 2).

**Table 2:** The differences on the distribution of plasmid replicons among *bla*_CHβLs_ positive and *bla*_CHβLs_ negtive *Enterobacter cloacae*

	*bla*_CHβLs_ positive strains (*n* = 106)	*bla*_CHβLs_ negtive strains (*n* = 166)	Chi-square	*P* value
IncCOI (*n* = 41)	21 (19.8%)	20 (12.4%)	3.046	0.081
IncFII (*n* = 62)	28 (26.4%)	34 (37.3%)	1.294	0.255
IncFIB (*n* = 58)	29 (27.4%)	29 (17.5%)	3.771	0.052
IncHI2 (*n* = 65)	37 (34.9%)	28 (16.9%)	11.574	0.001
IncHI2A (*n* = 64)	37 (57.5%)	27 (16.3%)	12.493	0.000
IncR (*n* = 27)	9 (8.5%)	19 (11.4%)	0.612	0.434

*CHβLs* Carbapenem-hydrolyzing β-lactamase

The distribution of *bla*_SHV_ was consistent with plasmid replicon IncR, and prevalence of *bla*_CTX-M_ was in accordance with the prevalence of IncFII, IncFIB and IncHI2A (*p* > 0.05). Additionally, the prevalence of oxacillin-hydrolyzing-*bla*_OXA_ and IncFIB as well as IncCOI was accordant (Table 3). Moreover, the prevalence of *bla*_KPC_ and *bla*_VIM_ were consistent with the distribution of IncCOI, IncFII, IncFIB, IncHI2 and IncHI2A, and no differences were observed on the distribution of *bla*_IMI_, *bla*_NDM_ and those of IncCOI, IncFII and IncFIB (*p* > 0.05) (Table 4).

**Table 3:** The differences on the distribution of plasmid replicons and resistant determinants among the β-lactamase producing *Enterobacter cloacae*

	*bla*_CMH_ (*n* = 130)	*bla*A_CT_ (*n* = 126)	*bla*_OXA_^a^ (*n* = 43)	*bla*_CTM-M_ (*n* = 47)	*bla*_SHV_ (*n* = 21)	*bla*_TEM_ (*n* = 90)
IncCOI (*n* = 48)	0.000	0.000	0.609	1.000	0.000	0.000
IncFII (*n* = 62)	0.000	0.000	0.037	0.137	0.000	0.012
IncFIB (*n* = 58)	0.000	0.000	0.146	0.272	0.000	0.041
IncHI2 (*n* = 65)	0.000	0.000	0.023	0.038	0.000	0.005
IncHI2A (*n* = 64)	0.000	0.000	0.031	0.053	0.000	0.003
IncR (*n* = 28)	0.000	0.000	0.006	0.007	0.347	0.000

^a^ Oxacillin-hydrolyzing-OXA

**Table 4:** The differences on the distribution of plasmid replicons and resistant determinants among the *bla*_CHβLs_ -carrying *Enterobacter cloacae*

Plasmid replicons	*bla*_KPC_ (*n* = 24)	*bla*_IMI_ (*n* = 17)	*bla*_VIM_ (*n* = 29)	*bla*_NDM_ (*n* = 22)
IncCOI (*n* = 21)	0.736	0.627	0.268	1.000
IncFII (*n* = 28)	0.652	0.080	1.000	0.451
IncFIB (*n* = 29)	0.551	0.058	1.000	0.337
IncHI2 (*n* = 37)	0.085	0.008	0.200	0.036
IncHI2A (*n* = 37)	0.085	0.008	0.200	0.036

*CHβLs* Carbapenem-hydrolyzing β-lactamase

## Discussion

β-lactamase is a primary resistance determinant, widely disseminating on mobile genetic elements across the opportunistic pathogens including *E. cloacae* [21]. Exploring the spread characteristics of β-lactamase among *E. cloacae* based on the global genome database of GenBank is quite important for illustrating the resistance characteristics of such strains and guiding rational drug use in clinic.

Our analysis showed that the number of *E. cloacae* has been continuously increasing since the genome of first one was submitted in 2003. More than 32 countries all over the world submitted the genomes, indicating the representativeness of these strains. To note, the host of these β-carrying *E. cloacae* strains were predominantly Homo sapiens, with the gastrointestinal tract being the major isolation resource, suggesting that Homo sapiens were the dominant host and gastrointestinal tract was predilection seat. Importantly, 106 *bla*_CHβLs_-carrying *E. cloacae* strains isolated during 2010–2020 were scattered among global 27 countries and 5 continents, indicating a rapid emergence and wide distribution of such strain, which alerts us the urgency of implementation of prevention and control measures.

Our analysis showed that *bla*_CMH_ was the most frequent β-lactamase gene. However, literature search with *bla*_CMH_ as the key word showed that *bla*_CMH-1_ was first detected in *E. cloacae* as a novel *bla*_AmpC_ gene at a Medical Center in Southern Taiwan [22]. Since then, *bla*_CMH-2_ and *bla*_CMH-3_ were sequentially identified in India and Europe [22, 23]. Thereafter, no *bla*_CMH_ was reported in PubMed database albeit genomic analysis showed the widest distribution of these enzyme. To our surprise, the most prevalent *bla*_CMH-6_, *bla*_CMH-4_, *bla*_CMH-5_ and *bla*_CMH-3_ were not identified among *E. cloacae* at all. Moreover, *bla*_ORN-1_, identified in the chromosome of *Raoultella. ornithinolytica* in 2004 [24, 25], has never been reported in *E. cloacae*. Interestingly, *bla*_CARB-2_ as a carbenicillin-hydrolyzing enzyme, has been identified within multiple strains including *Klebsiella. pneumonia* [26], *Achromobacter xylosoxidan* [27], *Escherichia. coli* [28], *Acinetobacter pittii* [29] and *E. cloacae* [30] in a variety of countries, however, was quite rare in our study. Which may be related to its clinical importance. *bla*_LAP-2_ as a narrow-spectrum β-lactamase was also rare in our study, albeit it has been reported [31] [32]. Furthermore, *bla*_SCO-1_ was a novel plasmid-mediated class A β-lactamase with carbenicillinase characteristics in *E. coli* [33], has not been reported in *E. cloacae* until now. As we know that *bla*_ACT_ was also a plasmid-encoded *ampC* gene [34]*.* Although the prevalence of *bla*_ACT_ was secondary to *bla*_CMH_ in our study, distribution of exact *bla*_ACT_-variants was not so high. Note worthily, the most common *bla*_ACT-59_ in our study has never been reported. Which may be due to the limitation of screening methods. It was reported that *bla*_VEB-3_ was encoded by the chromosome and located in an integron, and only 2 *bla*_VEB-2_ genes were detected in our study. However, outbreak of infection caused by *bla*_VEB-3_-carrying-*E. cloacae* has been reported in China [35],

Additionally, *bla*_KPC_, *bla*_VIM_, *bla*_NDM_, *bla*_IMI_ and *bla*_IMP_ were the major *bla*_CHβLs_ accounting for carbapenem resistance in global *E. cloacae*. Among them, *bla*_KPC-2_ and *bla*_VIM-4_ were the most predominant ones. Which is a light different from previous report showing *bla*_KPC-2_ and *bla*_IMP-8_ was the main *bla*_CHβLs_ within *E. cloacae* in China [36]. Noteworthily, 28 *bla*_VIM-4_-carrying *E. cloacae* ST873 were only found in Homo sapiens in France, indicating that there was a clonal dissemination of such strain among Homo sapiens in France during 2010–2020, which was not reported previously, albeit nosocomial infections caused by *E. cloacae* ST873 in 2 hospitals in France has been reported [37]. As a novel *bla*_CHβLs_, *bla*_FLC-1_ belongs to Ambler class A β-lactamases, has been identified an *E. cloacae* Complex isolated from food products [38]. Interestingly, such enzyme displayed a distinctive substrate profile, hydrolyzing penicillin, narrow- and broad-spectrum cephalosporins, aztreonam, and carbapenems but not extended-spectrum cephalosporin. In addition, *bla*_NMC-A_, a class A *bla*_CHβL_, has been frequently detected in *E. cloacae* [39, 40] [41, 42], albeit we just found 2 *bla*_NMC-A_ in this study. As *bla*_CHβLs_, *bla*_GES-24_ seems to have a broader host than *bla*_GES-2_ although we only found 2 *bla*_GES-2_ and 1 *bla*_GES-24_ in this study. To date, all reports on *bla*_IMI-1_ focus on *E. cloacae*, indicating that *E. cloacae* may be the best host for *bla*_IMI_.

The higher prevalence of *bla*_TEM_ and *bla*_SHV_ among *bla*_CHβLs_-carriers in our study was in accordance with a previous report to some degree, which showed that *bla*_CTX-M_, *bla*_TEM_ and *bla*_SHV_ were mostly detected concurrently with *bla*_CHβLs_ [43]. Albeit no distribution difference of *bla*_CTX-M_ was observed. Notably, the significantly higher distribution of *bla*_CMH_ among non-CHβLs-producers may indicate that *bla*_CMH_ may be the predominant gene conferring β-lactams among the strains without *bla*_CHβLs._

Furthermore, the multiple STs identified in our study displayed a genetic diversity of β-lactamase producing *E. cloacae*. It seemed that clonal dissemination for such strain was rare except for *bla*_VIM-4_-carrying ST873 ones, suggesting that the spread of CREL was mainly mediated by mobile elements such as plasmids.

Additionally, variously distinct plasmid replicons detected in our study indicate their dissemination potential for resistant determinants. Noteworthily, the obviously higher prevalence of IncHI2 and IncHI2A among *bla*_CHβLs_-carrying strains may suggest association between *bla*_CHβLs_ and IncHI2. It was reported that IncHI2 widely detected in global CRE genomes, was termed as 'super-plasmids' resulting from the large size and prolific carriage of resistance determinants [44]. And the consistent distribution of such plasmid and *bla*_KPC_ and *bla*_VIM_ may indicate a good cost fitness between them.

There were several limitations in this study. First, the number of *E. cloacae* was relatively small which may result from the reason that *E. cloacae* was only little part of *E. cloacae* complex. Second, the resistance profiles of these strains were not available for us to compare the difference between the genotypes and phenotypes. Some of the strain information were missing, which was not beneficial for us to fully illustrate the characterization of *E. cloacae*. Third, some new enzymes are devoid of further phenotypic descriptions because they were directly obtained from whole-genome sequencing studies. Anyway, it is currently difficult to draw an accurate global picture of this bacteria, highlighting the need for more comprehensive genome sequence data and genomic analysis.

In summary, almost all the *E. cloacae* contained β-lactamase encoding gene. Among the global *E. cloacae*, *bla*_CMH_ and *bla*_ACT_ were main *bla*_AmpC_ genes. *Bla*_TEM_ and *bla*_CTX-M_ were the predominant ESBLs. *Bla*_KPC_, *bla*_VIM_ and *bla*_NDM_ were the major CHβLs. Additionally, diversely distinct STs and different replicons were identified.

## Supplementary Information

**Additional file 1.**

## Data Availability

The datasets used and/or analyzed during the current study are available from GenBank and the accession number and web link to datasets for the provided name of these strains were shown in table S1.xlsx.

## Abbreviations

CRE: Carbapenem-resistant Enterobacteriaceae
ESBLs: Extended-spectrum β-lactamases
CHβLs: Carbapenem-hydrolyzing β-lactamase
pAmpCs: Plasmid-mediated AmpC β-lactamases
CREL: Carbapenem resistance *Klebsiella pneumoniae*
KPC: *Klebsiella** pneumoniae* Carbapenemase
NDM: New Delhi metallo-beta-lactamase
ESBLs: Extended-spectrum β-lactamases
MLST: Multi-locus sequence typing
STs: Sequence types
