# Supplementary material for: Molecular characteristics of global β-lactamase-producing Enterobacter cloacae by genomic analysis
Source: BMC Microbiol. 2022 Oct 21;22:255. doi: 10.1186/s12866-022-02667-y (PMC9585713; doi:10.1186/s12866-022-02667-y)
Supplement: Supplementary file 1 — Additional file 1. [file 12866_2022_2667_MOESM1_ESM.doc]

**Supporting Information**

**Molecular characteristics of global β-lactamase-producing *Enterobacter cloacae* by genomic analysis**

Jincao Hu^1#^, Jia Li^1#^, Chang Liu^1^, Yan Zhang^1^, Hui Xie^1^, Chuchu Li^2^, Han Shen^1*^, Xiaoli Cao^1*^

Author Affiliations:

1. Department of Laboratory Medicine, Nanjing Drum Tower Hospital, the affiliated Hospital of Nanjing University Medical School, Nanjing, Jiangsu, China
2. Department of Acute Infectious Disease Control and Prevention, Jiangsu Provincial Center for Disease Control and Prevention, Nanjing, China.

Jincao Hu and Jia Li contribute equally to the work

Corresponding author

Xiaoli Cao, PH. D

Associate chief technician

Zhongshan Road 321, Gulou, Nanjing, Jiangsu Province, PR China.

Tel: 00 86 25 83105360

E-mail address: [cao-xiao-li@163.com](mailto:cao-xiao-li@163.com)

Co-corresponding author

Han Shen,

Chief technician

Zhongshan Road 321, Gulou, Nanjing, Jiangsu Province, PR China.


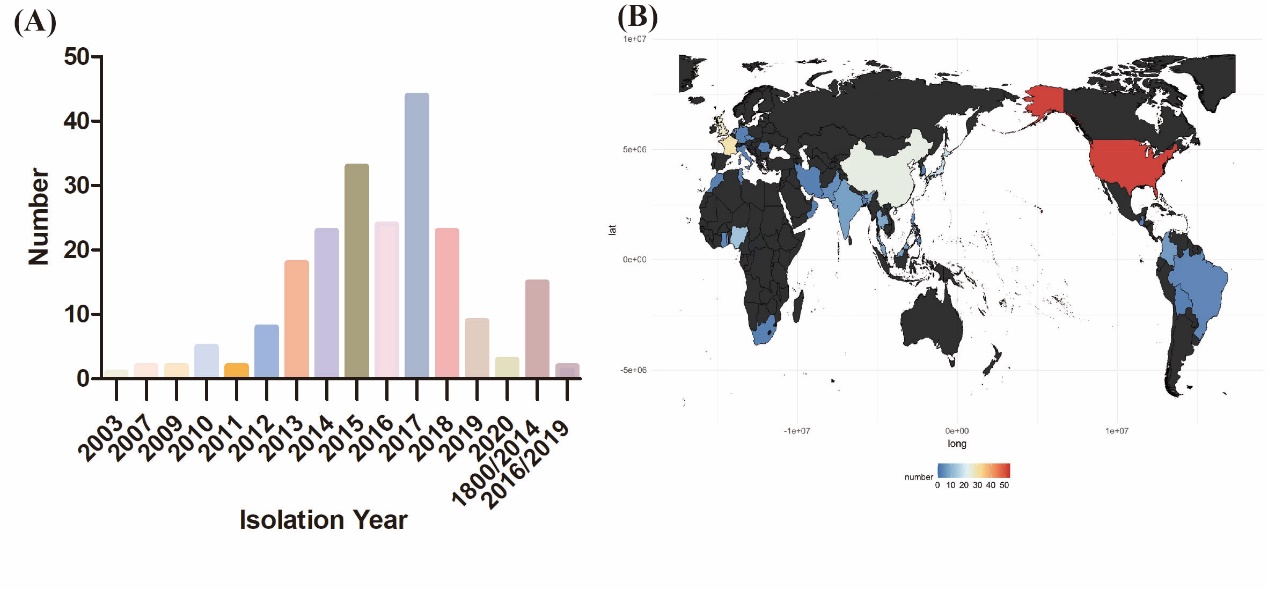


**Figure S1.** The number of global *Enterobacter cloacae*strains collected each year and the distribution of the *Enterobacter cloacae* submitted by different countries. (A) The number of global *Enterobacter cloacae*strains collected each year. (B) The distribution of the *Enterobacter cloacae* submitted by different countries. The redder the color, the higher the value, and the closer the color is to green, the lower the value.

**Table S1.** The accession number and web link to datasets for the provided name of these strains (Double-click to open the Microsoft Excel worksheet below and view the full information).
